# Barriers and Facilitators of Psychological Help-Seeking of People With Depression, Anxiety, and Stress Symptoms Among ASEAN Countries: A Systematic Review

**DOI:** 10.1177/00207640251367289

**Published:** 2025-09-10

**Authors:** Diany U. Syafitri, Shofia Mawaddah, Jennifer Y. F. Lau, June S. L. Brown

**Affiliations:** 1 Department of Psychology at the Institute of Psychiatry, Psychology, and Neuroscience, King’s College London, UK; 2 Faculty of Psychology, Universitas Islam Sultan Agung, Semarang, Indonesia; 3 Faculty of Educational Sciences, Universitas Negeri Medan, Sumatera Utara, Indonesia; 4 Centre for Psychiatry and Mental Health, Wolfson Institute of Population Health, Queen Mary University of London, UK

**Keywords:** help-seeking, formal psychological help, mental health, common mental disorder, Southeast Asia

## Abstract

**Background:**

Despite growing rates of common mental health disorders among country members of the Association of Southeast Asia Nations (ASEAN), there continue to be treatment gaps in these countries.

**Aim:**

To systematically identify and synthesise barriers and facilitators to accessing formal psychological help for common mental health disorders in the existing literature.

**Methods:**

APA PsycINFO, Web of Science, Scopus, and the Indonesian Portal Garuda were searched for studies reporting barriers or facilitators to individuals’ engaging in formal psychological help-seeking for common mental health disorders in country members of ASEAN. Participants in the studies were aged 18 years and above and included both quantitative and qualitative designs. Results were reported using PRISMA guidelines. Due to the heterogeneity of data and study designs, a narrative synthesis was chosen as an analysis strategy.

**Results:**

This review included forty-six studies. The barriers to formal psychological help-seeking were (1) social and cultural factors, which comprised of stigma, sociocultural and religious, and treatment-related issues, (2) personal factors, such as low mental health knowledge, self-reliance and disclosure difficulty, and (3) structural factors related to the low availability and affordability of mental health services. The facilitators were (1) social and cultural factors related to social and religious aspects, (2) personal factors comprising personal motivation, the presence and severity of mental health problems, and mental health literacy, and (3) structural factors such as accessibility and the system of mental health services, as well as gender differences.

**Conclusions:**

Social, cultural, personal, and structural factors are essential for formal help-seeking in ASEAN. Future studies and intervention development might examine these factors further.

## Introduction

The Association of Southeast Asian Nations (ASEAN) comprises 10 countries: Indonesia, Malaysia, Singapore, Thailand, Vietnam, Cambodia, the Lao People’s Democratic Republic, the Philippines, Myanmar, and Brunei Darussalam. This association aims to foster partnerships in social, cultural, economic, and other fields and to promote regional peace among the countries (ASEAN Secretariat, 2024a). According to the ASEAN Secretariat (2023) ASEAN population, the ASEAN population totalled over 670 million, with the 20 to 54 years age group comprising the largest share (50.6%), followed by the 5 to 19 years age group (24.4%) in 2023. Despite being located in similar regional areas, ASEAN member states (AMS) exhibit considerable diversity. The population varies widely; Indonesia has the largest population, with more than 280 million, while Brunei Darussalam has about 450.000 people. The education, political, and legal systems are also varied. Singapore has the highest Gross Domestic Product (GDP), making it 1 of the 10 wealthiest countries in the world. In contrast, Lao and Myanmar have the least, highlighting the wealth disparities among AMS (ASEAN Secretariat, 2024b). The total rural population in ASEAN comprised 46.35%, with considerable diversity. Singapore has 0% of its land area classified as rural, while Cambodia has 74.4% (World Bank, 2025). ASEAN has more than 1,500 tribes and 1,200 local languages. Among the largest ethnic groups are the Javanese (14.96%), Vietnamese (12.77%), Thai (9.24%), Sundanese (5.94%), Malays (5.09%), and Chinese (5.00%). Muslims are the largest religious group, accounting for 38.8%, followed by Buddhists at 25.4% and Christians at 20.2% (Moorthy & Benny, 2012). Approximately 80% of the population identifies with one of these religions (Pew Research Center, 2023).

Among AMS, mental health disorders such as depression and anxiety are common. The World Health Organisation (WHO) reported more than 22 and 19 million cases of anxiety and depression from all AMS (World Health Organization, 2017). For anxiety, Lao and the Philippines have the lowest, and Malaysia has the highest prevalence. Indonesia had the highest cases of anxiety and depression. Several countries in this region have surpassed the 4.4% and 3.6% global prevalence of depression and anxiety (World Health Organization, 2017). Untreated common mental disorders could lead to worse psychiatric outcomes (Kisely et al., 2006), such as the persistence of disorders, comorbidity, substance abuse, and suicide attempts (Henriksen et al., 2015).

Despite the growing number of mental health problems, only a small number of individuals with mental health difficulties seek treatment in the ASEAN countries. In Vietnam, only 5% of people with mental distress sought help (Giang et al., 2010), while in Indonesia, only 9% of people diagnosed with depression received professional help (Health Research and Development Agency Indonesian Ministry of Health, 2018). Similarly, only 11% of people with mental health problems received treatment in Thailand (ASEAN Secretariat, 2016). A slightly higher proportion, only 21.4% of Singaporeans (Subramaniam et al., 2020) and 20% of Malaysians with a mental disorder, sought professional help (Raaj et al., 2021). The gap between needing and accessing treatments may arise from the low intention of people with mental health problems to seek help from professionals (Dessauvagie et al., 2022; Seera et al., 2020). The ASEAN Secretariat (2016) mental health system report mentioned that challenges to seeking formal psychological help are misconceptions of the cause of mental illness, lack of knowledge of mental health, stigma, lack of social support to access formal help, and low accessibility and affordability of services.

In addition to structural factors, given the sociocultural diversity in the region, the cultural and religious factors may also influence the decision to seek formal psychological help. Culture plays a significant role in all aspects of mental health, from the recognition, development, and expression of distress to coping styles, help-seeking options, and treatments (Hwang et al., 2008; Kleinman, 1980). Explanatory models provide culturally relevant explanations for the underlying causes of distress and illness and strategies for achieving health and wellness in group and individual cultural contexts (Saint-Arnault, 2009). According to Hofstede’s cultural dimension model, the AMSs were characterised as a group-oriented culture (Chi Anh et al., 2015). The people in this cultural group (e.g. Asians) are commonly reported to exhibit a somatic expression of distress due to discouraged emotional expression (Chun et al., 1996), which may be related to deeper philosophical or cultural stances that reinforce the mind-body-spirit view (Hwang et al., 2006). The models also influence help-seeking options, including the availability of social resources, where family is often seen as the primary source of support, and it is expected to seek help only from group members (Saint-Arnault, 2009).

Similar to culture, religion also offers alternative perspectives on the meaning of suffering and its treatment (Wamser et al., 2011). In addition to low familiarity with the professionals and more accessibility to the religious sources, the perceived different belief systems between the clients who have religious beliefs and the mental health professionals might pose a barrier to seeking help (Abe-Kim et al., 2004). However, studies further found that the influence of religion towards help-seeking relies on particular aspects of religiosity, such as a high level of religiousness, religious involvement, and conservatism, which have been shown to increase the probability of seeking help from informal, religious-based sources (Abe-Kim et al., 2004; Hays & Lincoln, 2017; Moreno et al., 2022).

The most recent scoping review published in Asia-Pacific Psychiatry of barriers and facilitators of mental health services for mental disorders among ASEAN countries has illuminated that stigma, low literacy, cultural, and system were the most prominent barriers, and social support, outreach programmes, self-awareness, and accessibility were the primary facilitators (Andary et al., 2023). This review has provided a helpful map of barriers and facilitators of mental health service utilisation by focussing on general mental health issues (e.g. psychosis, substance abuse, and people with HIV) and from diverse perspectives of stakeholders (e.g. service users, policymakers, and caregivers). However, studies found that there were different pathways and factors affecting help-seeking for severe mental illness (SMI; e.g. psychosis), such as help-seeking timeline and sources of help, which related to perceived symptom severity (Skrobinska et al., 2024), compared to common mental disorders. In some contexts, there was a tendency to rely on others and informal sources to seek help among people with SMI (Ibrahim Awaad et al., 2022; Sadath et al., 2014). Unfortunately, the review did not separate the views by type of participant.

Following the previous review’s results and noting the importance of focussing on specific types of mental disorders and their stakeholders, this study deems it necessary to identify barriers and facilitators to formal help-seeking for common mental disorders to develop more specific recommendations. Therefore, this study aims to systematically identify and synthesise the existing literature on barriers and facilitators to formal psychological help-seeking among adults in ASEAN from the perspective of service users.

## Method

### Scope of the Study

The review followed the guidance of Preferred Reporting Items for Systematic Reviews and Meta-Analyses (PRISMA) 2020 (Page et al., 2021). This study only included 7 out of 10 Southeast Asian countries, leaving out Myanmar, Lao, and Brunei. This decision was made because there were few or no studies on seeking mental health help from these three countries, as reported in the WHO Mental Health Atlas (World Health Organization, 2021) and Google Scholar. This study included papers published in English and Indonesian and searched the local Indonesian database Portal Garuda. The protocol of this systematic review has been registered at the PROSPERO in the York Centre of Reviews and Dissemination (ID CRD42023434784).

### Database and Search Strategy

Four electronic databases (APA PsycINFO, Web of Science, Scopus, and Indonesian Portal Garuda) were searched by DS and SM from May to June 2023. The search terms used aimed to represent the concept “facilitators and barriers” of “help-seeking” for “mental health problems” in seven Southeast Asian countries. Synonyms of each search term were generated using thesaurus and terminologies used in previous review studies. Medical Subject Headings (MeSH) were also applied to the PsycINFO database. Boolean operators of OR and AND and truncation were applied for the free text search. In addition, a citation search was also implemented. The complete search terms are displayed in Supplemental Appendix 1.

### Eligibility Criteria

The inclusion criteria of study participants were: (1) adults with a mean age of 18 years and above or where at least 80% of participants were aged 18 years and above; (2) resided in ASEAN countries at the time of the study but if a study included multiple countries, only data from the seven included countries were extracted; (3) were diagnosed/not diagnosed with common mental health disorders (depression, anxiety, and stress) but where study reported multiple mental disorders, it was included if it has at least 80% participants with common mental disorders. Studies also had to (4) address facilitators or barriers in a formal help-seeking setting; (5) be published in English and Indonesian language; (6) no date restrictions applied; (7) be published in a peer-reviewed journal; and (8) conducted with either qualitative, quantitative, and/or mixed-method designs.

In this study, facilitators are factors that facilitate, promote, and support the seeking of formal psychological help, while barriers prevent or obstruct the process (W. Shi et al., 2020). The participants in this review range from general non-clinical populations to populations with current or previous common mental health disorders such as stress, depression, and anxiety. Formal psychological help-seeking is the proactive search for help for mental health issues through counselling, psychotherapy, and other psychological treatments delivered by trained mental health professionals (W. Shi et al., 2020).

The following are exclusion criteria: (1) Southeast Asian participants who resided in other countries; (2) participants who do not have mental health-related issues (e.g. medical and academic problems unless specifically related to mental health); (3) literature review studies; (4) studies that were not from the perspective of mental health service users (e.g. mental health professional’s or caregiver’s perspective); (5) grey literature and unpublished studies (e.g. conference proceedings, theses, and dissertations).

### Data Extraction and Risk of Bias Assessment

Each of the included full-text studies was given a study identification number. The data extracted from each paper consisted of (a) study information, (b) study design, (c) participants’ information, (d) mental health issues, (e) type of mental health services, and (f) results (variables or factors mentioned as barriers and/or facilitators). The extracted data from Indonesian papers were translated into English by the first reviewer (DS) and then re-checked by the second reviewer (SM).

The included study designs were quantitative (cross-sectional and longitudinal), quantitative with descriptive analysis, qualitative, and mixed methods. Data were extracted from the participants’ top 3 to 5 answers for quantitative studies with descriptive analysis of questionnaire responses on help-seeking. For qualitative studies, data were extracted from themes reflecting barriers or facilitators. The quantitative analysis reported inferential statistics of cross-sectional and longitudinal data, correlations and odds/hazard ratios between help-seeking and the independent variables were extracted (with at least *p* < .05). Studies with mixed-method data were extracted and categorised independently.

The Mixed Method Appraisal Tool (MMAT) was used to assess the risk of bias in this study (Hong et al., 2018). Two reviewers (DS and SM) independently assessed the risk of bias. In the assessment process, the two reviewers discussed any discrepancies found until an agreement was reached.

### Analysis Strategy

Due to the heterogeneity of study designs and help-seeking constructs, the narrative synthesis was considered the most acceptable analysis strategy (Aveyard et al., 2021; Boland et al., 2017). This strategy encompassed the textual descriptions of each study’s results, grouping studies based on particular attributes, tabulation to provide details of each study’s characteristics, translating data using thematic analysis for qualitative and quantitative studies, and subgroup analysis to explore relationships (Popay et al., 2006). The quantitative and qualitative studies were analysed separately, and the results were synthesised across the study designs.

The thematic analysis in this study followed the guidance outlined in Thomas and Harden (2008), which started with line-by-line coding of the extracted data from studies using NVIVO 14 software, creating the descriptive and analytical theme. The variable labels were extracted similarly to the conceptual themes from qualitative studies for the quantitative studies (Popay et al., 2006). The authors prepared two major themes relevant to the review question: barriers and facilitators of help-seeking. For quantitative cross-sectional and longitudinal studies, the variables (e.g. mental health literacy) with a positive direction of relationship or prediction to help-seeking will be categorised into facilitator themes and vice versa. For descriptive and qualitative studies, participants’ answers reflecting support for help-seeking will be categorised as facilitator and vice versa.

The first reviewer (DS) coded all the extracted data and then met with the second reviewer to re-check the assigned codes and group them into themes and subthemes. All reviewers then discussed and re-checked the codes, sub-themes, and themes to generate the analytic themes in the analysis process.

## Results

### Search Results

**Figure 1.**
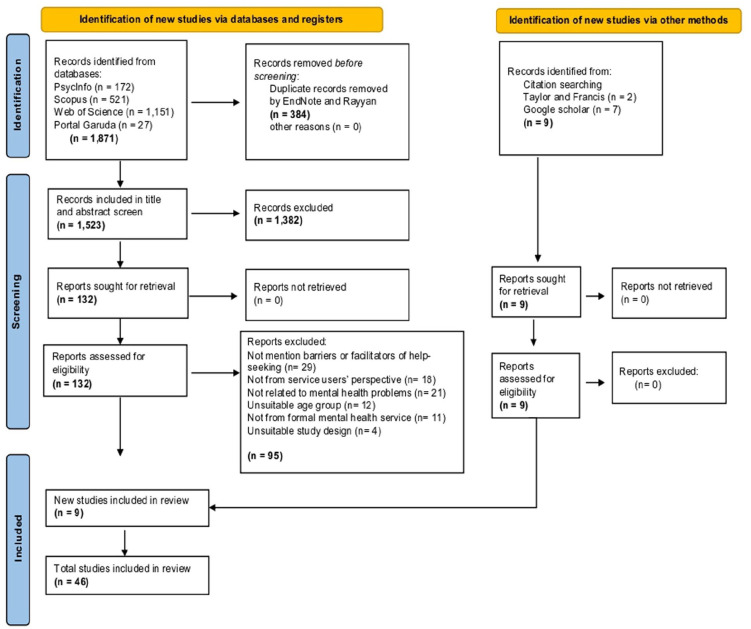
PRISMA flowchart.

Two reviewers (DS and SM) searched the databases. The initial search yielded 1,880 studies. After de-duplication using EndNote and Rayyan, 1,523 studies remained. DS and SM then independently screened the title and abstract against the predefined inclusion criteria, resulting in 132 studies eligible for full-text screening, with an additional 9 studies from another method, citation chaining (totalling 141). After obtaining the full-text version and independent screening, the final 46 studies were included in the review, with a publication date range of 2006 to 2023. Throughout the screening process, any disagreement between the two reviewers (DS and SM) was solved by discussion and involving opinions from the third and fourth reviewers (JB and JL) until agreement was achieved. The complete study selection process is presented in the PRISMA flowchart (Figure 1).

### Quality of the Studies

The risk of bias assessment showed that among the qualitative studies, 8 had strong quality, and 2 had low quality; for the quantitative studies, 15 had strong quality, 13 had moderate quality, and 6 had low quality, and both mixed-method and longitudinal studies had strong quality. Among four low-quality quantitative studies, the issues were mainly related to the sampling method, no justification of the statistical analysis, and not properly reporting the psychometric properties of the measurement tools. Among the two low-quality qualitative studies, the issues related to the unclear data analysis process. None of the studies were excluded based on these results. The complete results of the risk of bias assessment are displayed in Supplemental Appendix 2.

### Study Characteristics

The 46 included studies comprised 1 mixed-method study, 10 qualitative studies, 35 quantitative studies, comprising 3 with both cross-sectional and descriptive designs, 7 descriptive studies, 27 cross-sectional studies, and 1 longitudinal study. The sample sizes for qualitative studies ranged from 7 to 90 participants, while for quantitative studies, the sizes ranged from 77 to 8,205. The longitudinal study included 1,642 participants. The total number of participants included in this review is 44,867, comprising 25,738 females, 18,124 males, and 11 studies did not report gender. Additionally, 22 participants were categorised as others. The participants ranged in age from 18 to 70+ years old, with a mean age of 19.7 to 69.9 years.

Studies derived from Indonesia (*n* = 16), Singapore (*n* = 10), Malaysia (*n* = 8), Vietnam (*n* = 6), Thailand (*n* = 3), Philippines (*n* = 3), and Cambodia (*n* = 1). The majority were university students (*n* = 23), general populations (*n* = 14), outpatient (*n* = 5), and special populations (*n* = 4). Most settings were university-based, population-based, epidemiological studies, and healthcare-based. Most studies examined offline help-seeking, with only three on online. Help-seeking constructs included help-seeking attitude (*n* = 14), behaviour (*n* = 14), intention (*n* = 6), willingness (*n* = 3), and not specified (*n* = 9). The complete study characteristics are displayed in Tables 1–3.

**Table 1.** Study Characteristics Quantitative Design.

#ID	Study location	Authors	Methodology	Participant Characteristics	Help-seeking construct	Service provider	Risk of Bias assessment results
1	Indonesia	Nurdiyanto et al. (2023)	Cross-sectional online survey with Indonesian version of Attitude Toward Seeking Professional Psychological-Short Form scale	The general population from 3 ethnicities in Indonesia (Javanese, Sudanese, Minahasan), 590 participants, female 440, male 150, age range 15–50, mean age 24.26	Attitude	mental health professionals	Moderate quality
2	Indonesia	Rasyida (2019)	Cross-sectional online survey with Willingness to seek professional counselling outside the university (WSPCO) Scale	First and second-year undergraduate students, 205 participants, female 169, male 36 (age not reported)	Willingness	counsellor outside university	Moderate quality
3	Indonesia	Nisa and Syafitri (2022)	Cross-sectional online survey with Indonesian version of Attitude Toward Seeking Professional Psychological scale, Depression Anxiety Stress Scale (DASS), and Autonomy Scale	University students, 365 participants, female 211, male 154, age 18–21 (mean age not reported)	Attitude	Mental health professionals	Moderate quality
4	Indonesia	Dewi et al. (2022)	Cross-sectional online survey with Indonesian version of Depression Literacy (D-Lit), Self-Stigma of Mental Health (SSOH), Mental Health Seeking Attitude Scale (MHSAS)	University students, 353 participants, female 302, male 51, age 18–40 (mean age not reported)	Attitude	Mental health professionals	Low quality
5	Indonesia	Kartikasari and Ariana (2019)	Cross-sectional online survey with Indonesian version of Mental Health Seeking Help Intentions Scale (MHSIS), Mental Health Knowledge Schedule (MAKS), Self-Stigma of Seeking Help (SSOSH)	The general population, 571 participants, gender not reported, age 18–29 (mean age not reported)	Intention	Mental health professionals	Low quality
6	Indonesia	Maya (2021)	A cross-sectional survey with the Indonesian version of Multicomponent Mental Health Literacy Measure (MMHLM), Perceived-Devaluation Discrimination, and Attitude Toward Seeking Professional Psychological-Short Form (ATSPPH-SF)	The general population, had 349 participants, female 271, male 78, aged 18–24 (mean age not reported)	Attitude	Mental health professionals	Low quality
7	Indonesia	Falasifah and Syafitri (2021)	Cross-sectional online survey with Indonesian version of Attitude Toward Seeking Professional Psychological-Short Form (ATSPPH-SF), Mental Health Literacy Scale, Stigma Scale for Receiving Psychological Help (SSRPH)	*Mahasantri* (university student who lives in Islamic based dormitory), 175 participants, female 141, male 34, age 18–21 (mean age not reported)	Attitude	Mental health professionals	Moderate quality
8	Indonesia	Shabrina et al. (2022)	Cross-sectional online survey with demographic data, Indonesian version of the Big Five Inventory (BFI), and Mental Health Seeking Help Intentions Scale (MHSIS)	Undergraduate students, 480 participants, female 327, male 153, age 18 and above (mean age 20.18, *SD* 1.34)	Intention	Mental health professionals	Moderate quality
9	Indonesia	Putri et al. (2022)	Cross-sectional online survey with Indonesian version of Attitudes Toward Seeking Professional Psychological Help Scale Short Form (ATSPPH-SF), Multicomponent Mental Health Literacy Measure (MMHLM), Locus of Control Scale	Undergraduate students, 318 participants, female 191, male 127, aged 18–25	Attitude	Mental health professionals	Low quality
10	Indonesia	Syafitri and Rahmah (2021)	Cross-sectional online and offline surveys with the Indonesian Psychological Measure of Islamic Religiosity (I-PMIR), Islamic Religious Coping (RCOPE), Mental Health Seeking Help Intentions Scale (MHSIS), Attitudes Toward Seeking Professional Psychological Help Scale (ATSPPH)	University students, 731 participants, female 539, male 192, aged 17–26	Attitude and intention	Mental health professionals	Strong quality
11	Indonesia	Syafitri and Kusumaningsih (2021)	Cross-sectional offline survey with Indonesian version of Self-Concealment Scale, Disclosure Expectation Scale, Self-Stigma of Seeking Help Scale, and Online/Offline Counselling Attitude Scale	Undergraduate students, 756 participants, female 524, male 232, aged 18–20	Attitude	Mental health professionals	Strong quality
12	Indonesia	Setiawan (2006)	Cross-sectional offline survey with Indonesian version of The Willingness to Seek Counselling Questionnaire and questionnaire to identify factors discouraging/encouraging students from seeking counselling	Undergraduate students, 1279 participants, 846 male, 432 female, one unreported, age 17–26, mean age 19.7	Willingness	University counselling centre	Moderate quality
13	Malaysia	Khan et al. (2009)	Cross-sectional offline survey with a 25-item questionnaire to explore public attitudes towards, complications of, and preventive measures for depression and delays in seeking help	The general population, 1149 participants, female 704, male 445, aged 18 and above 50 (mean age 30, *SD* 11.15)	Attitude	Medical/mental health service provider	Strong quality
14	Malaysia	Ibrahim et al. (2019)	Cross-sectional offline survey with Malaysian version of General help-seeking questionnaire (GHSQ), Mental help-seeking attitude scale (MHSAS), Self-stigma of seeking help scale (SSOSH)	University students from low income, 77 participants (gender not mentioned), age 18–25 (mean age not reported)	Attitude	Mental health professionals	Moderate quality
15	Malaysia	Chai et al. (2021)	Cross-sectional offline survey with a sociodemographic questionnaire, the Malaysian version of the Patient Health Questionnaire (PHQ-9), three items related to help-seeking	Older adults, 273 participants, female 143, male 130, age 60 and above (mean age 69.9, *SD* 6.9)	Intention	Mental health professionals	Strong quality
16	Malaysia	Yeap and Low (2009)	Cross-sectional offline interview with (1) Respondent demographical profile, (2) A 10-item knowledge scale, (3) A nine-item attitude scale, and (4) One item of help-seeking tendency and reason for not seeking help	The general population, 587 participants, female 266, male 321, aged 18-above (mean age 33.9, *SD* 12.13)	Attitude	Mental health professionals	Strong quality
17	Malaysia	Aida et al. (2010)	Cross-sectional offline survey with sociodemographic items, Depression Anxiety Stress Scale (DASS-21), and questions on help-seeking behaviour	University students, 380 participants, female 221, male 159, age 20–25 (mean age not reported)	Behaviour	Counsellor, psychiatrists	Low quality
18	Malaysia	Pheng et al. (2019)	Cross-sectional offline survey with Stigma Scale for Receiving Psychological Help (SSRPH), Perceptions of Stigmatisation by Others for Seeking Help Scale (PSOSH), Self-Stigma of Seeking Help Scale (SSOHS), Beliefs About Psychological Services Scale (BAPS)	Undergraduate students, 327 participants (gender and age not reported)	Attitude	Counsellor	Moderate quality
19	Philippines	Clough et al. (2019)	Cross-sectional online survey with demographic information questionnaire, e-mental health concerns and barriers with two questions related to problems and barriers of e-mental health	The general population of 110 participants, female 71, male 39, aged 18–80, and a mean of age 24.83	Not specified	Mental health online service provider	Moderate quality
20	Singapore	Picco et al. (2016)	Population-based cross-sectional epidemiological offline survey with Attitudes Toward Seeking Professional Psychological Help 10-item	The general population, 3006 participants, female 1506, male 1500, aged 18–65, with a mean of age 40.9	Attitude	Mental health professionals	Strong quality
21	Singapore	Ng et al. (2008)	The population-based cross-sectional epidemiological survey, offline interview with a sociodemographic questionnaire, General Health Questionnaire-12, Schedules for Clinical Assessment in Neuropsychiatry (SCAN), and question-related help-seeking experiences	The general population, 2,801 participants, female 1,758, male 1043, aged 20–59, mean age 41, *SD* 9.6	Behaviour	Mental health professionals	Strong quality
22	Singapore	Shafie et al. (2021)	Population-based cross-sectional epidemiological offline survey with computer-assisted personal interviews (CAPI) Composite International Diagnostic Interview version 3.0 (WHO-CIDI 3.0) help-seeking related questions	The general population, 6,126 participants, female 3,058, male 3,068, aged 18 and above, mean age of 45.2	Behaviour	Mental health professionals	Strong quality
23	Singapore	J. Y. Shi and Kim (2020)	The cross-sectional online survey measured attitudes, injunctive norms, descriptive norms, self-efficacy, perceived risk, and behavioural seeking counselling	University students, 232 participants, female 144, male 88, age 18–28 (mean age 21.34, *SD* 1.75)	Behaviour	Counsellor	Moderate quality
24	Singapore	J. Y. Shi et al. (2020)	Cross-sectional online survey with the Short-Form 36 Health Survey Questionnaire (SF-36) and Theory of Planned Behaviour Questionnaire	University students, 232 participants, female 144, male 88, mean age 21.34	Intention	Counsellor	Moderate quality
25	Singapore	Subramaniam et al. (2020)	The population-based cross-sectional epidemiological offline survey, Face-to-face interviews with World Mental Health Composite International Diagnostic Interview (WMH‑CIDI) for mood, anxiety, and alcohol abuse disorders, Sheehan Disability Scale, Treatment Gap (Barriers to seeking care were elicited from the “services” module of the WMH-CIDI)	The general population, 6,126 participants, female 3,058, male 3,068, aged 18 and above, mean age of 45.2	Behaviour	Mental health professionals	Strong quality
26	Singapore	Chong et al. (2012)	The population-based cross-sectional epidemiological offline survey, Face-to-face interviews at the respondents’ homes with Composite International Diagnostic Interview (CIDI 3.0) Diagnostic Section for anxiety, mood, and alcohol abuse disorders, clinical severity (Sheehan’s Disability Scale), CIDI 3.0 service section	General populations, 6,616 participants, female 3,317, male 3,299, age 18 and above 65, mean age 42, *SD* 14.5	Behaviour	Mental health professionals	Strong quality
27	Thailand	Pumpuang et al. (2018)	Cross-sectional offline survey with a demographic questionnaire and Thai version of the Professional Psychological Help-seeking Questionnaire	University students, 343 participants, female 336, male 7, age 18 and above (mean age not reported)	Intention	Mental health professionals	Strong quality
28	Thailand	Seera et al. (2020)	Cross-sectional online survey with sociodemographic questionnaire, General Help-Seeking Questionnaire (GHSQ), General Health Questionnaire (GHQ-12), and Mental illness clinician attitude (MICA)	University students, 311 participants, female 155, male 145, age 18 and above, mean age 20.7	Behaviour	Mental health professionals	Strong quality
29	Vietnam	Tran-Chi et al. (2021)	Cross-sectional online survey with the Vietnamese version of Attitudes Towards Seeking Professional Psychological Help Short Form (ATSPPH – SF), Self-Concealment Scale (SCS), and COVID-19 Stress Scale (CSS)	University students, 478 participants, female 389, male 89 (age and mean age not reported)	Attitude	Mental health professionals	Moderate quality
30	Vietnam	Vu et al. (2023)	Cross-sectional face-to-face interview with the Vietnamese version of the Patient Health Questionnaire (PHQ-9) and General Anxiety Disorders Scale (GAD-7), World Health Organization Disability Assessment Schedule (WHODAS 2.0), Barriers to mental health access (The BACE-3)	Cancer patients with depression problems, 300 participants, female 157, male 143, aged 40–59 (mean age not reported)	Not specified	Mental health professionals	Strong quality
31	Vietnam	Van et al. (2021)	Cross-sectional offline survey with Perceived Barriers to Psychological Treatment (PBPT) scale, demographic, demand for mental health, PHQ 9, Multidimensional Scale of Perceived Social Support (MSPSS)	Older adults, 376 participants, female 250, male 126, age 60-above 70 (mean age not reported)	Not specified	Mental health professionals	Strong quality
32	Vietnam	Kamimura et al. (2018)	Cross-sectional offline survey with a questionnaire related to perceptions of mental illness and individuals with mental illness	University students, 533 participants, female 405, male 128, age 18-30, mean age 20.2, SD 1.7	Not specified	Mental health professionals	Moderate quality
33	Vietnam	Tien et al. (2021)	A cross-sectional survey, face-to-face and online, with a demographic questionnaire, the Vietnamese version of the Patient Health Questionnaire (PHQ-9), Generalised Anxiety Disorder (GAD-7), Academic Motivation Scale, and questions related to help-seeking	University students, 8,205 participants, female 6,028, male 2,147, other 22, mean age 20.5	Behaviour	Mental health professionals	Strong quality
34	Vietnam	Pham et al. (2020)	Cross-sectional offline survey with the Vietnamese version of the Inventory of Attitudes toward Seeking Mental Health Services (IASMHS) and Intention to Seek Professional Mental Healthcare	University students, 108 participants, female 70, male 38, age 18-above 45	Intention	Mental health professionals	Low quality

**Table 2.** Study Characteristics Qualitative Design.

#ID	Study location	Authors	Methodology	Participant characteristics	Help-seeking construct	Service provider	Risk of bias assessment results
35	Indonesia	Munira et al. (2023)	In-depth interview using an online messenger application, Beck Depression Inventory-II	Outpatient individuals with common mental health disorders diagnosis (depression, anxiety, and bipolar), 90 participants, female 82, male 8, age 18–32 (mean age not reported)	Behaviour	Mental health professionals in clinic/hospital	Strong quality
36	Indonesia	Putri et al. (2021)	online survey for participants’ recruitment, followed by individual semi-structured face-to-face interviews, Patient Health Questionnaire (PHQ) -9	Mental health service user (had a previous diagnosis of depression and anxiety), 8 participants, female 7, male 1, age 20-35 (mean age not reported)	Behaviour	Psychologists and psychiatrists in private and public practices	Strong quality
37	Indonesia	Biladina (2021)	Face-to-face structured interview	The general population, 25 participants, gender not reported, age 18–24	Not specified	Mental health professionals	Low quality
38	Indonesia	Lucia (2022)	Individual in-depth interview	University students (first- to third-year undergraduate students), 8 participants (gender and age not specified)	Not specified	Not specified	Low quality
39	Malaysia	Zay Hta et al. (2021)	Online individual semi-structured interview	LGBTQ+ individuals, 28 participants, age 21-34 (mean age 27, SD 3.77)	Behaviour	Counsellors, psychologist, and psychiatrist	Strong quality
40	Malaysia	Berry et al. (2020)	Prodromal Questionnaire and Strengths and Difficulties Questionnaire (SDQ), offline individual semi-structured interview	Vulnerable young people living in low-income areas, 9 participants, female 4, male 5, age 16-23, mean age 19.78, SD 2.86	Not specified	Mental health service at NGO	Strong quality
41	Philippines	Dela Cruz et al. (2023)	Individual semi-structured interview, Depression, Anxiety, Stress Scale (DASS)-21	The general population with online counselling experience, 11 participants, 9 female, 2 male, age 20–32 (mean age not reported)	Behaviour	Online mental health counselling	Strong quality
42	Singapore	Salamanca-Sanabria et al. (2023)	Online focus group discussion	University students, 30 participants, female 19, male 11, age 21–34, mean age 22.74, *SD* 1.36	Not specified	Mental health professionals in university (offline and online)	Strong quality
43	Singapore	Samari et al. (2022)	Face-to-face individual semi-structured interview,	In outpatient clinics with depressive disorder diagnosis, 33 participants, 18 female, 15 male, age 18-35, mean age 26, SD 4.6	Not specified	Mental health professionals	Strong quality
44	Singapore	Seow et al. (2021)	Offline individual in-depth interview	Outpatient psychotherapy with depression and anxiety diagnosis, 15 participants, 9 female, 6 male, age 21–65	Behaviour	Psychiatrist/psychologist at the Institute of Mental Health, a tertiary psychiatric hospital	Strong quality

**Table 3.** Study Characteristics Longitudinal Study and Mixed Method.

#ID	Study location	Authors	Methodology	Participant characteristics	Help-seeking construct	Service provider	Risk of bias assessment results
45	Thailand	Chiddaycha and Wainipitapong (2021)	Longitudinal study All medical students enrolled in the 2014-2019 mental health data were collected with Thai Mental Health Indicator 66 (TMHI-66). Data analysis was extracted from separate and confidential files of the faculty counselling centre.	First- to sixth-year medical students at university, 1642 participants, female 773, male 869, age 18 and above	Behaviour	University counselling centre	Strong quality
46	Cambodia and Philippines	Ho et al. (2018)	Mixed-method Offline survey and Focus Group Discussion with (a) demographic questionnaire, (b) Depression Literacy Scale (D-Lit), (c) Attitudes and Understanding Towards Mental Disorders (AUM), and (d) Attitudes Toward Seeking Professional Psychological Help-Short Form (ATSPPH-SF)	General populations Quantitative study: Cambodia 150 (female 114, male 36), Philippines 156 (female 71, male 85) Qualitative study: Cambodia 20 (15 female, 5 male), Philippines 19 (10 female, 10 male) Cambodia participants’ mean age for the quantitative study was 42.43, FGD 49.95 Philippines participants’ mean age for the quantitative study was 27.65, FGD 53.79	Attitude	Mental health professionals (not specified)	Strong quality

### Barriers to Formal Psychological Help-Seeking Across Study Designs

Table 4 summarises the barrier themes based on the number of studies, the study design, and the study identification number from which the themes were found. The prominent barriers below were reported in at least three studies and emerged in more than one type of study design.

**Table 4.** Barriers Themes.

Themes from qualitative studies	*N*	Study ID numbers	Themes from quantitative studies	*N*	Study ID numbers	Themes from descriptive studies	*N*	Study ID numbers
Stigma (mental illness stigma, public, and self-stigma)	9	35, 39, 42, 38, 37, 40, 36, 44, 36	Stigma (public and self-stigma)	4	18, 14, 11, 5	Low mental health knowledge (mental health problems and where to get treatments)	9	13, 2, 19, 30, 16, 25, 32, 26, 12
Treatment-related issues (lousy experience with mental health service, distrust of mental health service)	8	39, 36, 41, 37, 43, 40, 42, 38	Mental health problems (worse mental health conditions)	2	28, 24	Personal (self-reliance and disclosure difficulty)	7	13, 30, 25, 31, 32, 26, 12
Structure and system (availability of treatment, referral system, and affordability)	7	35, 36, 44, 39, 38, 41, 42	Demographic characteristics (middle education level, middle-income level, and older age group)	2	20, 22	Structure and system (financial concerns and transportation)	6	30, 16, 25, 31, 32, 12
Sociocultural (culture and religious beliefs, preference to seek help from significant others, unsupportive significant others)	6	41, 43, 46, 42, 36, 38	Personal (disclosure difficulty)	1	11	Stigma (public and self-stigma)	5	30, 16, 25, 31, 32
Low mental health knowledge (mental health problems and the treatments)	3	44, 39, 36	Sociocultural (being Indian and Malay)	1	20	Socio-religious (preference to seek help from significant others and religious belief)	5	2, 30, 16, 32, 12,
Personal (disclosure difficulty and self-reliance)	3	41, 42, 39				Treatment-related issues (doubting the effectiveness of mental health service)	3	19, 26, 12
Mental health problems	1	39						

#### Social and Cultural

##### Stigma

Stigma was found to be the most frequent barrier, totalling 18 studies. The quantitative studies (7) showed a significant relationship between stigma (public and self) towards attitude and intention to seek help, while the qualitative studies (11) mentioned similar findings.

##### Sociocultural and Religious

These themes were derived from six qualitative and five descriptive studies. The social factors relate to the preference to seek help from significant others and the significant others’ unsupportiveness of seeking professional help. The “grind culture” and specific religious beliefs were also found to be barriers.

##### Treatment-Related Issues

This theme was identified in the eight qualitative and three descriptive studies. Participants described a range of negative experiences with mental health services, such as distrust related to doubting confidentiality, the qualifications of mental health professionals, and scepticism of treatment effectiveness.

#### Personal

##### Low Mental Health Knowledge

This theme was found in nine descriptive and three qualitative studies. Most participants felt unsure of whom and where to seek help, were not aware of symptoms of mental health problems, and perceived that the problem was not severe enough to seek help.

##### Self-Reliance and Disclosure Difficulty

The personal themes were extracted from three qualitative and seven descriptive studies. The personal themes found were mainly the tendency of self-reliance and disclosure difficulty with professionals

#### Structure

##### Availability and Affordability of Mental Health Services

This theme emerged from seven qualitative and six descriptive studies. It illustrates the difficulties in accessing services, the lack of referrals among healthcare providers, and affordability.

### Facilitators to Formal Psychological Help-Seeking Across Study Designs

The facilitator themes are summarised in Table 5 according to the number of studies, the study design, and the study identification number that yielded the themes. The leading facilitators listed below appeared in more than one study design and were identified in at least three studies.

**Table 5.** Facilitators Themes.

Themes from qualitative studies	*N*	Study ID numbers	Themes from quantitative studies	*N*	Study ID numbers	Themes from descriptive studies	*N*	Study ID numbers
Personal motivation and perceived benefit of seeking help (internal motivation, personal past help-seeking experience, and preference for non-pharmacological treatment)	4	44, 35, 39, 41	Personal motivation and perceived benefit of seeking help	16	11, 3, 27, 23, 16, 24, 10, 34, 9, 28, 33, 15, 6, 8, 29, 21	Structure and system (adjusting counselling fee)	2	16, 12
Structure and system (accessibility, affordability, referral system, and social media campaign)	4	39, 35, 44, 41	Demographic characteristics (young age group, female, single, living alone, unemployed, and high and low education level)	10	16, 20, 1, 6, 8, 9, 22, 15, 25, 21, 33			
Social (support from significant others)	3	41, 35, 39	Mental health problems (indication and perceived severity of mental health problems)	7	21, 3, 17, 29, 33, 26, 45			
Perceived severity of mental health problems	2	40, 42	Social and religious (descriptive and subjective norm, religious coping, and others’ diagnosis of mental disorder)	5	23, 10, 4, 28, 27			
Treatment related-issues (non-judgemental mental health professionals and online counselling modality)	2	39, 41	Mental health literacy	5	46, 5, 6, 7, 9			
Mental health literacy	1	39	Pandemic COVID-19 situation	1	29			
Pandemic COVID-19 situation	1	41						

#### Social and Cultural

##### Social and Religious

These themes were derived from five quantitative and three qualitative studies. In the quantitative studies, subjective and descriptive norms, religious coping, and others’ diagnoses of mental disorders served as significant predictors of attitude and intention to seek help. The qualitative studies reported support from significant others to seek help.

#### Personal

##### Personal Motivation and Perceived Benefit of Seeking Help

THIS theme emerged from 16 quantitative and 4 qualitative studies. In quantitative studies, variables related to an individual’s internal capacity, such as self-efficacy, autonomy, personality traits, locus of control, the anticipated benefit of disclosure, perceived behavioural control, and a favourable attitude, were found to be significant predictors of help-seeking attitude and intention. Mental illness diagnosis and previous help-seeking were also significant predictors of seeking help. The qualitative studies repeatedly mentioned internal motivation to improve as a facilitator.

##### Mental Health Problems

This theme was found in six quantitative and two qualitative studies. The presence of stress, anxiety, and depression symptoms and perceived severity were found to serve as a significant predictor of help-seeking behaviour.

##### Mental Health Literacy

This theme was found in five quantitative and one qualitative study. Mental health literacy significantly predicts intention and attitudes towards seeking help.

#### Structural Factors

##### Demographic Characteristics

This theme is only derived from quantitative studies (10). Several demographic variables, such as female, divorced/unmarried, education level, and unemployment, significantly predict help-seeking.

##### Structure and System

This theme was found in four qualitative and two descriptive studies. Both types of studies describe affordability, accessibility, referrals from other healthcare providers, and social media campaigns for mental health as facilitators of help-seeking.

## Discussion

This review included studies from 2006 to 2023, indicating that help-seeking for mental health problems has recently garnered attention from scholars in ASEAN. However, based on the early scoping search and the WHO Mental Health Atlas, studies on this topic were not found in Lao, Myanmar, and Brunei. As mentioned, the AMS was diverse in its political, economic, and social aspects, contributing to the development of each country’s mental health system, policy, and research.

The barriers to seeking help in this review are generally consistent with those in other review studies among Asian populations, such as stigma, social and cultural factors, mental health knowledge, and systems. However, one difference was the personal motivation and perceived benefit of seeking help theme, which has rarely been identified as a common facilitator among other Asian populations. The most frequent facilitators found among Asians were social support (Andary et al., 2023), perceived distress (Martinez et al., 2020), adequate mental health knowledge (Munawar et al., 2022), and trust in mental health professionals (Choudhry et al., 2021).

## Social and Cultural Factors

### Stigma

Southeast Asian societies had strong roots in collectivism, where individuals are expected to behave according to cultural norms, and anything that is viewed as inappropriate or outside the norm, encompassing mental health conditions, will be seen as unacceptable and thus vulnerable to stigmatisation (Abdullah & Brown, 2011). Studies have shown that Asian Americans tend to have a higher stigma towards mental health disorders and seeking help than Whites (Tse & Haslam, 2021), African Americans, and Latino Americans (Cheng et al., 2013). The result is also consistent with the meta-analysis by Clement et al. (2015), which found that internalised and treatment stigma served as the fourth most endorsed barrier to help-seeking. In contrast, a study in the European Union showed that social and individual stigmatising attitudes were associated with increased willingness to seek help from formal services (Mojtabai, 2010), indicating cultural differences in stigma (Yang et al., 2007).

### Social Support

Sociocultural factors were found to be substantial barriers and, at the same time, facilitators to seeking help. On the one hand, the barriers from social factors were related to the tendency to seek help from significant others and the lack of social encouragement to access formal psychological help. Asian populations have long been known as a group-oriented social system, which focusses on the family as the primary source of support for the individual (Saint-Arnault, 2009). As they discourage openly expressing emotion to others (Shin, 2002), they generally do not value seeking help for mental health issues (Kung, 2003).

On the other hand, the support from significant others to access help and their favourable experiences using mental health services appeared to be significant facilitators of seeking formal help. Support from others in accessing help was also observed among Asian Americans, African Americans, and Arabs (Khatib et al., 2023; S. B. Kim & Lee, 2022; Planey et al., 2019). These results highlight the importance of social factors among collectivistic populations and how the quality of social support affects help-seeking.

### Treatment-Related Issues

Pertinent to the cultural context, as a collectivistic culture discourages emotional expression to other than family, the lack of trust towards mental health professionals is prevalent among Asians (Ho et al., 2018; Jorm et al., 2005), and they have a vital need for confidentiality in treatment (Sugiura et al., 2000). Previous studies found that Saudi Arabians and Chinese reported having a negative experience with formal mental health services that hindered them from seeking further help (Noorwali et al., 2022; W. Shi et al., 2020).

## Personal Factors

### Personal Motivation

Personal factors, including personal motivation, knowledge of mental health, and perceived severity of mental health problems, emerged as significant barriers and facilitators to seeking help. Studies found the prominence of internal motivation in seeking help, where motivation to improve was associated with more positive psychological help-seeking attitudes (Barta & Kiropoulos, 2023; Grosso et al., 2013; Randell et al., 2011).

### Self-Reliance

In the Southeast Asian context, this barrier might relate to cultural values. Asians are inclined to hold negative beliefs about disclosing personal matters and exercising self-control, which increases their likelihood of solving problems by themselves (B. S. K. Kim et al., 2001). Self-reliance, as the most frequent barrier to seeking help, was also found among university students in the United Kingdom and the United States (Lui et al., 2024).

### Mental Health Literacy

Low mental health knowledge, specifically related to insufficient knowledge of mental health services and low perceived mental health symptoms, was identified as a notable barrier and at the same time, mental health literacy came out as one of the facilitators. Studies in this review reported a significant but small to moderate relationship between mental health literacy and attitude and intention to seek help, consistent with a recent meta-analysis (Ozparlak et al., 2023). Consistent with previous studies, among Malaysians and Pakistanis, mental health knowledge also served as a prominent factor in help-seeking (Choudhry et al., 2021; Munawar et al., 2022).

### Mental Health Problems and Perceived Severity

Previous studies in the United Kingdom (Walters et al., 2008) and Australia (Edwards & Crisp, 2020) showed that distress severity was positively associated with service use (Rudell et al., 2008) . Considering the low mental health knowledge among Southeast Asians, it becomes exceedingly challenging to evaluate mental health problems’ severity.

## Structure and Demographic Factors

### Mental Health System and Facility

Given the Southeast Asian context, some countries in this region have no mental health policies and limited mental health facilities, and most are not integrated with community-based services (ASEAN Secretariat, 2016). Previous studies have consistently mentioned the structure and system as barriers and reported them as crucial in accessing formal psychological help (Reynders et al., 2014). Other studies in Saudi Arabia (Noorwali et al., 2022) and China (W. Shi et al., 2020) also reported similar findings.

### Gender

Various studies have documented that being female increases the likelihood of seeking help in different countries (S. B. Kim & Lee, 2022) because females generally have higher psychological openness (Mackenzie et al., 2006).

## Implications and Recommendations

This review features several gaps in the literature for future studies. First, among the studies with general populations and university students, most participants had no mental disorder diagnosis and were asked about their help-seeking preference if they were in distress. As these studies have highlighted the non-clinical populations’ help-seeking, future studies might consider focusing more on participants with mental disorder diagnoses and the actual service utilisation to better understand the factors influencing the behaviours of people who have sought help. Studies with specialised segments (e.g. adolescents) are also necessary as they are often considered more at risk of mental health problems.

Second, most of the studies identified were quantitative cross-sectional, with only one longitudinal study. Thus, it is challenging to demonstrate causation between help-seeking and the influencing variables. Future research might consider a longitudinal study design to examine how the help-seeking process plays out.

Third, this review indicated that help-seeking in ASEAN is highly influenced by cultural values of a collectivistic orientation. Future works might explore these cultural influences in depth. Developing an intervention targeting help-seeking in these populations might incorporate cultural values to increase its feasibility and acceptability.

Fourth, as personal motivation was identified as the most mentioned facilitator, future studies might examine personal factors and their relation to help-seeking. Intervention development might also focus on enhancing personal capacities.

Fifth, stigma and low mental health knowledge were identified as significant barriers to accessing mental health services. Interventions to improve mental health literacy might help reduce the stigma and improve help-seeking. Some programmes have been culturally adapted and tested for feasibility in increasing mental health literacy in Indonesia (Brooks et al., 2021) and Singapore (Tay, 2022). Further studies might focus on further developing and evaluating such programmes.

### Strengths and Limitations

First, this review tried to comprehensively describe existing literature on mental health help-seeking in Southeast Asia. Second, this review aimed to give a broad view of the literature on help-seeking by including all help-seeking constructs (attitude, intention, and behaviour). Although it might be helpful, the barriers and facilitators might differ in each construct. Third, the participants in this review range from young to older adults. While this might be beneficial in summarising the facilitators and barriers through generations, the pattern and factors of help-seeking might differ. Fourth, including university and population-based studies and clinical and non-clinical participants might be useful in depicting general facilitators and barriers to formal psychological help, regardless of the setting and participants’ condition. However, it also poses the possibility of overgeneralisation, as different types of participants and settings may have different barriers and facilitators. Fifth, this review employed the counts of themes based on the number of studies to illustrate the findings. Although the count of themes is necessary to give insights into future research and develop interventions targeting help-seeking, there can be a tendency to overemphasise particular themes based on the counts. Sixth, further inferential statistics and meta-analysis could not be conducted due to the high heterogeneity of measurement tools used in the studies. Lastly, this review did not include grey literature or studies written in languages other than Indonesian and English. Using Indonesian has resulted in more studies on this language and country being included in the review. While this approach may introduce a risk of selection bias, it may also be advantageous in describing general ASEAN and Indonesia’s help-seeking patterns.

## Conclusions

The review findings highlight several barriers and facilitators of formal psychological help-seeking for common mental health problems among Southeast Asian populations. The most mentioned barriers are stigma, low mental health knowledge, treatment-related issues, structure and system, sociocultural and religious, and personal factors. On the other hand, personal motivation and perceived benefit of seeking help, specific demographic characteristics, structure and system, social and religious factors, mental health problems, and mental health literacy are the most cited facilitators. Cultural nuances were found to influence help-seeking among Southeast Asians highly.

## Supplemental Material

sj-docx-1-isp-10.1177_00207640251367289 – Supplemental material for Barriers and Facilitators of Psychological Help-Seeking of People With Depression, Anxiety, and Stress Symptoms Among ASEAN Countries: A Systematic Review Supplemental material, sj-docx-1-isp-10.1177_00207640251367289 for Barriers and Facilitators of Psychological Help-Seeking of People With Depression, Anxiety, and Stress Symptoms Among ASEAN Countries: A Systematic Review by Diany U. Syafitri, Shofia Mawaddah, Jennifer Y. F. Lau and June S. L. Brown in International Journal of Social Psychiatry

sj-docx-2-isp-10.1177_00207640251367289 – Supplemental material for Barriers and Facilitators of Psychological Help-Seeking of People With Depression, Anxiety, and Stress Symptoms Among ASEAN Countries: A Systematic Review Supplemental material, sj-docx-2-isp-10.1177_00207640251367289 for Barriers and Facilitators of Psychological Help-Seeking of People With Depression, Anxiety, and Stress Symptoms Among ASEAN Countries: A Systematic Review by Diany U. Syafitri, Shofia Mawaddah, Jennifer Y. F. Lau and June S. L. Brown in International Journal of Social Psychiatry
