# Supplementary material for: Barriers and Facilitators of Psychological Help-Seeking of People With Depression, Anxiety, and Stress Symptoms Among ASEAN Countries: A Systematic Review
Source: Int J Soc Psychiatry. 2025 Sep 10;72(3):419–38. doi: 10.1177/00207640251367289 (PMC13121814; doi:10.1177/00207640251367289)
Supplement: sj-docx-1-isp-10.1177_00207640251367289 – Supplemental material for Barriers and Facilitators of Psychological Help-Seeking of People With Depression, Anxiety, and Stress Symptoms Among ASEAN Countries: A Systematic Review [file sj-docx-1-isp-10.1177_00207640251367289.docx]

**Appendix I: search terms**

**Search terms:**

| **Concept 1**  **Facilitators and barriers** | **AND** | **Concept 2**  **Mental health** | **AND** | **Concept 3**  **Help-seeking** | **AND** | **Concept 4**  **Southeast Asia** |
| --- | --- | --- | --- | --- | --- | --- |
| OR facilitator  OR promote  OR support  OR encourage  OR barriers  OR obstruct  OR hurdle |  | OR mental health  OR mental disorder  OR mental illness  OR psychiatric disorder  OR emotional problem  OR behaviour problem  OR anxiety  OR depression |  | OR help-seeking  OR seeking help  OR help-seeking behaviour  OR help-seeking behavior  OR treatment seeking  OR help-seeking intention  OR help-seeking attitude  OR care seeking  OR seeking care  OR service use  OR psychological help-seek  OR mental health service  OR counseling seeking  OR seeking counseling  OR counseling intention  OR counseling attitude |  | OR Indonesia  OR Malaysia  OR Singapore  OR Viet/VietNam  OR Thai/Thailand  OR Philippines  OR Filipino  OR Cambodia |

**Searh queries for APA PsycInfo (12 June 2023) 172 results**

**Search queries link:**

<https://access.ovid.com/custom/redirector/wayfless.html?idp=https://kclidpdev.kcl.ac.uk/idp/shibboleth&url=http://ovidsp.ovid.com/ovidweb.cgi?T=JS&NEWS=N&PAGE=main&SHAREDSEARCHID=lGLaYT4FfissbWrPczOS5ClUq8oui29MudvQzaWxGnYh94fGtL2Z1fy9MUeKa20m>

APA PsycInfo <1806 to June Week 1 2023>

1 facilitator*.mp. [mp=title, abstract, heading word, table of contents, key concepts, original title, tests & measures, mesh word] 19615

2 exp Treatment Barriers/ 7113

3 barriers.mp. [mp=title, abstract, heading word, table of contents, key concepts, original title, tests & measures, mesh word] 79194

4 promote*.mp. [mp=title, abstract, heading word, table of contents, key concepts, original title, tests & measures, mesh word] 145399

5 encourage*.mp. [mp=title, abstract, heading word, table of contents, key concepts, original title, tests & measures, mesh word] 79777

6 obstruct*.mp. [mp=title, abstract, heading word, table of contents, key concepts, original title, tests & measures, mesh word] 11296

7 hurdle*.mp. [mp=title, abstract, heading word, table of contents, key concepts, original title, tests & measures, mesh word] 2342

8 1 or 2 or 3 or 4 or 5 or 6 or 7 312367

9 mental health.mp. [mp=title, abstract, heading word, table of contents, key concepts, original title, tests & measures, mesh word] 280410

10 mental disorder*.mp. [mp=title, abstract, heading word, table of contents, key concepts, original title, tests & measures, mesh word] 197335

11 exp Mental Disorders/ 1024969

12 mental illness.mp. [mp=title, abstract, heading word, table of contents, key concepts, original title, tests & measures, mesh word] 51241

13 psychiatric disorder*.mp. [mp=title, abstract, heading word, table of contents, key concepts, original title, tests & measures, mesh word] 44291

14 emotional problem*.mp. [mp=title, abstract, heading word, table of contents, key concepts, original title, tests & measures, mesh word] 7833

15 exp Emotional Disturbances/ 10864

16 behavio$r problem*.mp. [mp=title, abstract, heading word, table of contents, key concepts, original title, tests & measures, mesh word] 44936

17 exp Behavior Problems/ 32560

18 anxiet*.mp. [mp=title, abstract, heading word, table of contents, key concepts, original title, tests & measures, mesh word] 288185

19 exp Anxiety/ 89304

20 depress*.mp. [mp=title, abstract, heading word, table of contents, key concepts, original title, tests & measures, mesh word] 429185

21 exp Suicide/ 31434

22 suicide.mp. [mp=title, abstract, heading word, table of contents, key concepts, original title, tests & measures, mesh word] 68358

23 self-harm.mp. [mp=title, abstract, heading word, table of contents, key concepts, original title, tests & measures, mesh word] 7727

24 exp Psychosis/ 128046

25 psycho*.mp. [mp=title, abstract, heading word, table of contents, key concepts, original title, tests & measures, mesh word] 1667091

26 9 or 10 or 11 or 12 or 13 or 14 or 15 or 16 or 17 or 18 or 19 or 20 or 21 or 22 or 23 or 24 or 25 2559019

27 "help-seek*".mp. [mp=title, abstract, heading word, table of contents, key concepts, original title, tests & measures, mesh word] 13204

28 seek* help.mp. [mp=title, abstract, heading word, table of contents, key concepts, original title, tests & measures, mesh word] 6649

29 help-seek* behavio$r*.mp. [mp=title, abstract, heading word, table of contents, key concepts, original title, tests & measures, mesh word] 8270

30 exp Help Seeking Behavior/ 16292

31 "treatment seek*".mp. [mp=title, abstract, heading word, table of contents, key concepts, original title, tests & measures, mesh word] 6135

32 help-seek* intention*.mp. [mp=title, abstract, heading word, table of contents, key concepts, original title, tests & measures, mesh word] 497

33 help-seek* attitude.mp. [mp=title, abstract, heading word, table of contents, key concepts, original title, tests & measures, mesh word] 55

34 care seek*.mp. [mp=title, abstract, heading word, table of contents, key concepts, original title, tests & measures, mesh word] 6848

35 seek* care.mp. [mp=title, abstract, heading word, table of contents, key concepts, original title, tests & measures, mesh word] 1672

36 psychological help-seek*.mp. [mp=title, abstract, heading word, table of contents, key concepts, original title, tests & measures, mesh word] 284

37 "service use".mp. [mp=title, abstract, heading word, table of contents, key concepts, original title, tests & measures, mesh word] 5850

38 mental health service*.mp. [mp=title, abstract, heading word, table of contents, key concepts, original title, tests & measures, mesh word] 75999

39 exp Mental Health Services/ 47432

40 exp Health Care Seeking Behavior/ 9927

41 counsel$ing seek*.mp. [mp=title, abstract, heading word, table of contents, key concepts, original title, tests & measures, mesh word] 36

42 seek* counsel$ing.mp. [mp=title, abstract, heading word, table of contents, key concepts, original title, tests & measures, mesh word] 669

43 counsel$ing attitude*.mp. [mp=title, abstract, heading word, table of contents, key concepts, original title, tests & measures, mesh word] 122

44 counsel$ing intention*.mp. [mp=title, abstract, heading word, table of contents, key concepts, original title, tests & measures, mesh word] 10

45 27 or 28 or 29 or 30 or 31 or 32 or 33 or 34 or 35 or 36 or 37 or 38 or 39 or 40 or 41 or 42 or 43 or 44 106578

46 exp Southeast Asian Cultural Groups/ 2199

47 "southeast asia".mp. [mp=title, abstract, heading word, table of contents, key concepts, original title, tests & measures, mesh word] 1238

48 indonesia*.mp. [mp=title, abstract, heading word, table of contents, key concepts, original title, tests & measures, mesh word] 4125

49 malaysia*.mp. [mp=title, abstract, heading word, table of contents, key concepts, original title, tests & measures, mesh word] 5220

50 singapore*.mp. [mp=title, abstract, heading word, table of contents, key concepts, original title, tests & measures, mesh word] 5759

51 viet*.mp. [mp=title, abstract, heading word, table of contents, key concepts, original title, tests & measures, mesh word] 8847

52 thai*.mp. [mp=title, abstract, heading word, table of contents, key concepts, original title, tests & measures, mesh word] 5882

53 philippines.mp. [mp=title, abstract, heading word, table of contents, key concepts, original title, tests & measures, mesh word] 3055

54 filipino*.mp. [mp=title, abstract, heading word, table of contents, key concepts, original title, tests & measures, mesh word] 2677

55 cambodia*.mp. [mp=title, abstract, heading word, table of contents, key concepts, original title, tests & measures, mesh word] 1587

56 46 or 47 or 48 or 49 or 50 or 51 or 52 or 53 or 54 or 55 34543

57 8 and 26 and 45 and 56 172

**Search queries for Web of Science (12 June 2023) 1,151 results**

**Search queries link:**

[**https://www.webofscience.com/wos/woscc/summary/2cb546c4-9da0-4b2d-bf69-257becbe51a1-8fc71f70/relevance/1**](https://www.webofscience.com/wos/woscc/summary/2cb546c4-9da0-4b2d-bf69-257becbe51a1-8fc71f70/relevance/1)

facilitator* OR promote OR encourage OR support OR barrier* OR obstruct OR hurdle (All Fields) and "mental health" OR "mental disorder*" OR "mental illness" OR "psychiatric disorder*" OR anxiet* OR depress* OR suicide OR "self-harm" OR psycho* OR "emotional problem*" OR "behavio$r problem" OR "emotional disturbance*" (All Fields) and help-seek* OR seek* help OR help-seek* behavio$r OR treatment seek* OR seek* treatment OR help-seek* intention* OR help-seek* attitude* OR care seek* OR seek* care OR "mental health service*" OR "psychological help-seek*" OR counsel$* seek* OR seek* counsel$* OR counsel$* attitude* OR counsel$* intention* (All Fields) and "southeast asia" OR indonesia* OR malaysia* OR singapore* OR viet* OR thai* OR philippines OR filipino* OR cambodia* (All Fields)

**Search queries for Scopus (12 June 2023) 517 results**

( ( TITLE-ABS-KEY ( facilitator* OR promote OR encourage OR support OR barrier* OR obstruct OR hurdle ) AND TITLE-ABS-KEY ( "mental health" OR "mental disorder*" OR "mental illness" OR "psychiatric disorder*" OR anxiet* OR depress* OR suicide OR "self-harm" OR psycho* OR "emotional problem*" OR "behavior$r problem" ) AND TITLE-ABS-KEY ( "help-seek*" OR "seek* help" OR "help-seek* behavio?r" OR "treatment W/1 seek*" OR "help-seek* intention*" OR "help-seek* attitude*" OR "care seek*" OR "seek* care" OR "mental health service*" OR "psychological help-seek*" OR "counsel?* seek*" OR "seek* counsel?*" OR "counsel?* attitude*" OR "counsel?* intention*" ) AND TITLE-ABS-KEY ( "southeast asia" OR indonesia* OR malaysia* OR singapore* OR viet* OR thai* OR philippines OR filipino* OR cambodia* ) ) )

**Portal Garuda (12 June 2023) 27 results**

Keywords searched: *bantuan psikologis, sikap mencari bantuan, mencari bantuan, pencarian pertolongan*
