# Supplementary material for: Barriers and Facilitators of Psychological Help-Seeking of People With Depression, Anxiety, and Stress Symptoms Among ASEAN Countries: A Systematic Review
Source: Int J Soc Psychiatry. 2025 Sep 10;72(3):419–38. doi: 10.1177/00207640251367289 (PMC13121814; doi:10.1177/00207640251367289)
Supplement: sj-docx-2-isp-10.1177_00207640251367289 – Supplemental material for Barriers and Facilitators of Psychological Help-Seeking of People With Depression, Anxiety, and Stress Symptoms Among ASEAN Countries: A Systematic Review [file sj-docx-2-isp-10.1177_00207640251367289.docx]

**Appendix II**

**QUANTITATIVE STUDIES-RISK OF BIAS ASSESSMENT USING MIXED METHOD APPRAISAL TOOL (MMAT) 2018 VERSION (QUANTITATIVE DESCRIPTIVE SECTION)**

Following MMAT guidelines, the domain of the study that meets the criteria was given a 20% score, so it was possible to calculate the overall score to determine the quality of the study. Studies that met 80-100% of the criteria of the MMAT were classified as having strong quality, 60% of criteria moderate quality, and under 40% low quality.

| **No** | **Country** | **Title** | **Authors** | **MMAT questions** | | | | | | | **Conclusion** | **SCORE** | **CATEGORY** | **CONCERNS** |
| --- | --- | --- | --- | --- | --- | --- | --- | --- | --- | --- | --- | --- | --- | --- |
|  |  |  |  | **a** | **b** | **1** | **2** | **3** | **4** | **5** |  |  |  |  |
| 1 | Indonesia | Analysis on the Attitude Towards Seeking Professional Psychological Help | Nurdiyanto et al., 2023 | YES | YES | YES | NO | YES | CT | YES | YES= 3, CAN'T TELL= 1, NO= 1 | 60 | Moderate quality |  |
| 2 | Indonesia | *Faktor yang menjadi hambatan untuk mencari bantuan psikologis formal di kalangan mahasiswa* Eng: Barriers of formal psychological help-seeking among university students | Annisa, 2019 | YES | YES | YES | CT | YES | CT | YES | YES= 3, CAN'T TELL= 2 | 60 | Moderate quality |  |
| 3 | Indonesia | *Hubungan antara distres psikologis dan kemandirian dengan sikap terhadap pencarian bantuan psikologis pada mahasiswa Universitas Islam Sultan Agung Semarang*  Eng: Relationship between psychological distress and autonomy with attitude towards seeking professional psychological help among students of Universitas Islam Sultan Agung Semarang | Nisa & Syafitri, 2022 | YES | YES | YES | CT | YES | CT | YES | YES= 3, CAN'T TELL= 2 | 60 | Moderate quality |  |
| 4 | Indonesia | *Hubungan antara literasi kesehatan mental depresi dan stigma diri dengan sikap mencari bantuan masalah kesehatan mental pada mahasiswa keperawatan di Institut Teknologi dan Kesehatan Bali*  Eng: Relationship between depression mental health literacy and self-stigma with attitude towards seeking mental health among nursing student in Bali Institute of Technology and Health | Dewi et al., 2022 | YES | YES | YES | CT | NO | CT | NO | YES= 1, CAN'T TELL= 2, NO= 2 | 20 | Low quality | sampling strategy, measurement tools, and statistical analysis |
| 5 | Indonesia | *Hubungan Antara Literasi Kesehatan Mental, Stigma Diri Terhadap Intensi Mencari Bantuan Pada Dewasa Awal* Eng: Relationship between mental health literacy and self-stigma towards intention to seek help among young adults | Kartikasari & Ariana, 2019 | YES | YES | NO | NO | YES | CT | NO | YES= 1, CAN'T TELL= 1, NO= 3 | 20 | Low quality | sampling strategy, sampling representativeness, and statistical analysis |
| 6 | Indonesia | *Kontribusi Literasi Kesehatan Mental dan Persepsi Stigma Publik terhadap Sikap Mencari Bantuan Profesional Psikologi*  Eng: Contribution of mental health literacy and public stigma perception towards attitude of seeking professional psychological help | Maya, 2021 | YES | YES | NO | CT | YES | CT | YES | YES= 2, CAN'T TELL= 3 | 40 | Low quality | sampling strategy |
| 7 | Indonesia | *Literasi Kesehatan Mental dan Stigma Publik Sebagai Prediktor Sikap Terhadap Bantuan Psikologis Pada Mahasantri* Eng: Mental health literacy and public stigma as predictors to attitude towards seeking psychological help among Islamic undergraduate students | Falasifah & Syafitri, 2021 | YES | YES | YES | CT | YES | CT | YES | YES= 3, CAN'T TELL= 2 | 60 | Moderate quality |  |
| 8 | Indonesia | Mental health help-seeking intentions: The role of personality traits in a sample of college students | Shabrina et al., 2022 | YES | YES | YES | CT | YES | CT | YES | YES= 3, CAN’T TELL= 2 | 60 | Moderate quality |  |
| 9 | Indonesia | *Peran literasi kesehatan mental, lokus kontrol, dan gender terhadap sikap mencari bantuan profesional psikologi pada mahasiswa* Eng: The Role of Mental Health Literacy, Locus of Control, and Gender on Attitudes Toward Seeking Professional Psychological Help of Undergraduate Students | Putri, 2022 | YES | YES | NO | CT | YES | CT | YES | YES= 2, CAN'T TELL= 2, NO= 1 | 40 | Low quality | sampling strategy |
| 10 | Indonesia | *Peran Religiusitas Dan Religiusitas Dalam Mencari Bantuan Psikologis Pada Mahasiswa di Semarang*  Eng: The Role of Religiousity and Religious Coping Towards Seeking Psychological Help Among College Students in Semarang | Syafitri & Rahmah, 2021 | YES | YES | YES | YES | YES | CT | YES | YES= 4, CAN'T TELL= 1 | 80 | Strong quality |  |
| 11 | Indonesia | *Sikap terhadap bantuan psikologis (tatap muka dan daring) ditinjau dari penyembunyian diri, harapan pengungkapan, dan stigma diri pada mahasiswa*  Eng: Attitudes toward psychological help (face-to-face and online) in  terms of self-concealment, disclosure expectations, and selfstigma among students | Syafitri & Kusumaningsih, 2021 | YES | YES | YES | YES | YES | CT | YES | YES= 4, CAN'T TELL= 1 | 80 | Strong quality |  |
| 12 | Indonesia | Willingness to seek counselling, and factors that facilitate and inhibit the seeking of counselling in Indonesian undergraduate students | Setiawan, 2006 | YES | YES | YES | CT | YES | CT | YES | YES= 3, CAN’T TELL= 2 | 60 | Moderate quality |  |
| 13 | Malaysia | Attitude toward depression, its complications, prevention and barriers to seeking help among ethnic groups in Penang, Malaysia | Khan et al., 2009 | YES | YES | YES | YES | YES | CT | YES | YES= 4, CAN'T TELL= 1 | 80 | Strong quality |  |
| 14 | Malaysia | Do depression literacy, mental illness beliefs and stigma influence mental health help-seeking attitude? A cross-sectional study of secondary school and university students from B40 households in Malaysia | Ibrahim et al., 2019 | YES | YES | YES | YES | YES | CT | YES | YES= 4, CAN'T TELL= 1 | 80 | Moderate quality |  |
| 15 | Malaysia | Intention to seek professional help for depression and its associated factors among elderly patients in tenkera health clinic, Melaka, Malaysia | Chai et al., 2021 | YES | YES | YES | YES | YES | CT | YES | YES= 4, CAN'T TELL= 1 | 80 | Strong quality |  |
| 16 | Malaysia | Mental health knowledge, attitude and help-seeking tendency: A Malaysian context | Yeap & Low, 2009 | YES | YES | YES | YES | YES | CT | YES | YES= 4, CAN'T TELL= 1 | 80 | Strong quality |  |
| 17 | Malaysia | Psychological disorders and help seeking behaviour among Malaysian medical students in their clinical years | Aida et al., 2014 | YES | YES | YES | CT | NO | CT | YES | YES= 2, CAN'T TELL= 2, NO= 1 | 40 | Low quality | measurement tools |
| 18 | Malaysia | Stigma and Attitudes Toward Seeking Counseling Among Undergraduate Students | Pheng et al., 2019 | YES | YES | CT | YES | YES | CT | YES | YES= 3, CAN'T TELL= 2 | 60 | Moderate quality |  |
| 19 | Philippines | Going global: do consumer preferences, attitudes, and barriers to using e-mental health services differ across countries? | Clough et al., 2019 | YES | YES | YES | CT | YES | CT | YES | YES= 3, CAN'T TELL= 2 | 60 | Moderate quality |  |
| 20 | Singapore | Attitudes Toward Seeking Professional Psychological Help: Factor Structure and Socio-Demographic Predictors | Picco et al., 2016 | YES | YES | YES | YES | YES | YES | YES | YES= 5 | 100 | Strong quality |  |
| 21 | Singapore | Health beliefs and help-seeking for depressive and anxiety disorders among urban Singaporean adults | Ng et al., 2008 | YES | YES | YES | YES | YES | CT | YES | YES= 4, CAN'T TELL= 1 | 80 | Strong quality |  |
| 22 | Singapore | Help-Seeking Patterns Among the General Population in Singapore: Results from the Singapore Mental Health Study 2016 | Shafie et al., 2021 | YES | YES | YES | YES | YES | CT | YES | YES= 4, CAN'T TELL= 1 | 80 | Strong quality |  |
| 23 | Singapore | Integrating risk perception attitude framework and the theory of planned behavior to predict mental health promotion behaviors among young adults | Shi & Kim, 2020 | YES | YES | YES | CT | YES | CT | YES | YES= 3, CAN'T TELL= 2 | 60 | Moderate quality |  |
| 24 | Singapore | Mental Health Conditions in Young Asian Adults and Motivation to Seek Counseling: A Cross-Country Study | Shi et al., 2020 | YES | YES | YES | CT | YES | CT | YES | YES= 3, CAN'T TELL= 2 | 60 | Moderate quality |  |
| 25 | Singapore | Minding the treatment gap: results of the Singapore Mental Health Study | Subramaniam, 2020 | YES | YES | YES | YES | YES | CT | YES | YES= 4, CAN'T TELL= 1 | 80 | Strong quality |  |
| 26 | Singapore | Where do people with mental disorders in Singapore go to for help? | Chong et al., 2012 | YES | YES | YES | YES | YES | CT | YES | YES= 4, CAN'T TELL= 1 | 80 | Strong quality |  |
| 27 | Thailand | Factors predicting intention among nursing students to seek professional psychological help | Pumpuang et al., 2018 | YES | YES | YES | YES | YES | CT | YES | YES= 4, CAN'T TELL= 1 | 80 | Strong quality |  |
| 28 | Thailand | Help-seeking behaviours for mental health problems in medical students: Studies in Thailand and India | Seera et al., 2020 | YES | YES | YES | YES | YES | CT | YES | YES= 4, CAN'T TELL= 1 | 80 | Strong quality |  |
| 29 | Vietnam | The Influence of COVID-19 Stress and Self-Concealment on Professional Help-Seeking Attitudes: A Cross-Sectional Study of University Students | Tran-Chi et al., 2021 | YES | YES | YES | CT | YES | CT | YES | YES= 3, CAN'T TELL= 2 | 60 | Moderate quality |  |
| 30 | Vietnam | Mental health, functional impairment, and barriers to mental health access among cancer patients in Vietnam | Vu et al., 2023 | YES | YES | YES | YES | YES | YES | YES | YES= 5 | 100 | Strong quality |  |
| 31 | Vietnam | Perceived Barriers to Mental Health Services among the Elderly in the Rural of Vietnam: A Cross Sectional Survey in 2019 | Van et al., 2021 | YES | YES | YES | YES | YES | CT | YES | YES= 4, CAN'T TELL= 1 | 80 | Strong quality |  |
| 32 | Vietnam | Perceptions of mental health and mental health services among college students in Vietnam and the United States | Kamimura et al., 2018 | YES | YES | YES | CT | YES | CT | YES | YES= 3, CAN'T TELL= 2 | 60 | Moderate quality |  |
| 33 | Vietnam | Utilization of mental health services among university students in Vietnam | Tien et al., 2021 | YES | YES | YES | YES | YES | CT | YES | YES= 4, CAN'T TELL= 1 | 80 | Strong quality |  |
| 34 | Vietnam | Understanding mental health services and help-seeking behaviours among college students in Vietnam | Pham et al., 2020 | YES | YES | NO | CT | YES | CT | YES | YES= 2, CAN'T TELL= 2, NO= 1 | 40 | Low quality | sampling strategy |

**QUALITATIVE STUDIES- RISK OF BIAS ASSESSMENT USING MIXED METHOD APPRAISAL TOOL (MMAT) 2018 VERSION (QUALITATIVE SECTION)**

| No | **Country** | **Title** | **Authors** | **MMAT questions** | | | | | | | **CONCLUSION** | **SCORE** | **CATEGORY** | **CONCERN** |
| --- | --- | --- | --- | --- | --- | --- | --- | --- | --- | --- | --- | --- | --- | --- |
|  |  |  |  | **a** | **b** | **1** | **2** | **3** | **4** | **5** |  |  |  |  |
| 1 | Indonesia | Barriers and facilitators to access mental health services among people with mental disorders in Indonesia: A qualitative study | Munira et al., 2023 | YES | YES | YES | NO | YES | YES | YES | YES=4, NO= 1 | 80 | High quality |  |
| 2 | Indonesia | Exploring the perceived challenges and support needs of Indonesian mental health stakeholders: a qualitative study | Putri et al., 2021 | YES | YES | YES | YES | YES | YES | YES | YES=5 | 100 | High quality |  |
| 3 | Indonesia | *Stigma related to Asking for Help from a Mental Health Professional in Bandung, Indonesia*  Eng: Stigma related to Asking for Help from a Mental Health Professional in Bandung, Indonesia | Biladina, 2021 | YES | YES | YES | CT | NO | YES | NO | YES=2, CAN’T TELL= 1, NO= 3 | 40 | Low quality | data analysis and coherence among data, analysis, and interpretation |
| 4 | Indonesia | *Gambaran Penyebab Mahasiswa Enggan Mencari Bantuan Profesional di Masa Pandemi Covid-19*  Eng: Descriptive Study of The Reason Behind College Students’ Reluctance to Seek Professional Help during the Covid-19 Pandemic | Hernawati, 2022 | YES | YES | YES | YES | NO | NO | NO | YES= 2, NO= 3 | 40 | Low quality | data analysis and coherence among data, analysis, and interpretation |
| 5 | Malaysia | Barriers and facilitators to professional mental health help-seeking behavior: Perspective of Malaysian LGBT individuals | Zay Hta et al., 2021 | YES | YES | YES | YES | YES | YES | YES | YES= 5 | 100 | High quality |  |
| 6 | Malaysia | Views of young people in Malaysia on mental health, help-seeking and unusual psychological experiences | Berry et al., 2020 | YES | YES | YES | YES | YES | YES | YES | YES= 5 | 100 | High quality |  |
| 7 | Philippines | "Hello, can you hear me?": Narratives of online mental health counselling among Filipino adults during the pandemic | Dela Cruz, 2022 | YES | YES | YES | YES | YES | YES | YES | YES= 5 | 100 | High quality |  |
| 8 | Singapore | Exploring the Perceptions of mHealth Interventions for the Prevention of Common Mental Disorders in University Students in Singapore: Qualitative Study | Salamanca-Sanabria et al., 2023 | YES | YES | YES | YES | YES | YES | YES | YES= 5 | 100 | High quality |  |
| 9 | Singapore | Perceived mental illness stigma among family and friends of young people with depression and its role in help-seeking: A qualitative inquiry | Samari et al., 2022 | YES | YES | YES | YES | YES | YES | YES | YES= 5 | 100 | High quality |  |
| 10 | Singapore | A Qualitative Approach to Understanding the Holistic Experience of Psychotherapy Among Clients | Seow et al., 2021 | YES | YES | YES | YES | YES | YES | YES | YES= 5 | 100 | High quality |  |

**COHORT STUDY-RISK OF BIAS ASSESSMENT USING MIXED METHOD APPRAISAL TOOL (MMAT) 2018 VERSION (QUANTITATIVE NON-RANDOMIZED SECTION)**

| **No** | **Country** | **Title** | **Authors** | **MMAT questions** | | | | | | | **Conclusion/Category** |
| --- | --- | --- | --- | --- | --- | --- | --- | --- | --- | --- | --- |
|  |  |  |  | **a** | **b** | **1** | **2** | **3** | **4** | **5** |  |
| 1 | Thailand | Mental health among Thai medical students: Preadmission evaluation and service utilisation | Chiddaycha & Wainipitapong, 2021 | YES | YES | YES | YES | YES | YES | YES | YES= 5/strong quality |

**MIXED METHOD STUDY-RISK OF BIAS ASSESSMENT USING MIXED METHOD APPRAISAL TOOL (MMAT) 2018 VERSION (MIXED METHOD SECTION)**

| **No** | **Country** | **Title** | **Authors** | **MMAT questions** | | | | | | | **Conclusion/Category** |
| --- | --- | --- | --- | --- | --- | --- | --- | --- | --- | --- | --- |
|  |  |  |  | **a** | **b** | **1** | **2** | **3** | **4** | **5** |  |
| 1 | Cambodia and Philippines | Depression literacy and health-seeking attitudes in the Western Pacific region: a mixed-methods study | Ho et al., 2018 | YES | YES | YES | YES | YES | YES | YES | YES= 5/ strong quality |
